# Willingness-to-Pay-Associated Right Prefrontal Activation During a Single, Real Use of Cosmetics as Revealed by Functional Near-Infrared Spectroscopy

**DOI:** 10.3389/fnhum.2019.00016

**Published:** 2019-02-04

**Authors:** Keith Kawabata Duncan, Tatsuya Tokuda, Chiho Sato, Keiko Tagai, Ippeita Dan

**Affiliations:** ^1^Shiseido Global Innovation Center, Yokohama, Japan; ^2^Applied Cognitive Neuroscience Laboratory, Chuo University, Tokyo, Japan

**Keywords:** applied neuroscience, neuroeconomics, neuroergonomics, willingness-to-pay, fNIRS, cosmetics, DLPFC, prefrontal cortex

## Abstract

Use of applied neuroscience to complement traditional methods of consumer research is increasing. Previously, fMRI has shown that prefrontal activity contains information relating to willingness-to-pay (WTP). The aim of the present study was to determine if functional near infrared spectroscopy (fNIRS) can record WTP-related brain activation in the dorsolateral prefrontal cortex (DLPFC) during a single, real use of cosmetic products. Thirty female participants, were divided into two groups (one low frequency users of foundation and one high frequency users of foundation), asked to apply different foundations to their face and then record how much money they were willing to pay. The oxyhemoglobin time series was analyzed with the GLM and the correlation between the beta scores for the foundations and their respective WTP values conducted for each participant. These subject level correlations were then converted to z scores and averaged for each group. The results revealed a significant mean correlation for the high but not low frequency group. In other words, the brain activity in right hemisphere dorsolateral PFC (RH-DLPFC) during single, real use of foundations correlated with their respective WTP values for the high frequency but not low frequency group. The difference between groups may reflect the importance of learning and automation on activity in RH-DLPFC. Our research provides further evidence supporting the use of fNIRS to complement traditional consumer research in a commercial setting and to extend neuroscience research into more naturalistic environments.

## Introduction

The use of neuroscience to complement consumer research using traditional self-report continues to grow in popularity (Lee et al., 2007, 2017), and is expected to hold an important position in the area of neuroergonomics (Curtin and Ayaz, 2018). This growth has been fueled by the hope that as brain data does not rely on self-report or conscious behavioral responses, it will be less influenced by cognitive biases, fabrication et cetera and further catalyzed by a number of findings which have found a link between brain activity with a particular neural structure and some commercially relevant outcome such as purchase behavior (Plassmann et al., 2007; Berns and Moore, 2012; Misawa et al., 2014; Venkatraman et al., 2014). For example, Plassmann et al. (2007) demonstrated that activity in right hemisphere medial orbitofrontal cortex (RH-mOFC) and RH dorsolateral prefrontal cortex (RH-DLPFC), recorded with fMRI, correlates with willingness-to-pay (WTP), which is the maximum amount of money that a consumer is willing to part with to obtain a product. The correlation of mOFC and WTP may arise due to the role of mOFC in the encoding of hedonic value of experience (Kringelbach et al., 2003; Plassmann et al., 2007). There are a number of functions that have been associated with RH-DLPFC. Among them, its role in working memory (Owen et al., 2005; Rottschy et al., 2012; Barbey et al., 2013; Nee et al., 2013) may be important in the context of evaluation of hedonic experience. Hence, the activity within these structures may provide a complementary source of valuable information regarding consumer product experience and evaluation.

However, due to the constraints inherent in fMRI, such as prohibition of metal, limitation in movement and so on, the neural response of consumers to a product is often tested using a photograph of the product, rather than during use of the product itself (Plassmann et al., 2007). This may be suboptimal for two reasons. First, although consumers can make purchase decisions based on information, including photographs, provided by the company selling the product, and expert and non-expert reviews, consumers often prefer to have a real experience of a product before purchase either in store or online (Skrovan, 2017). In particular for cosmetics such as foundation, the consumer’s decision to purchase can be assisted by testing the product in order to assess the suitability with respect to the shade, coverage and texture. Second, for an item which is experienced multimodally, brain activity in response to a photograph or even mental visualization may differ from brain activity in response to real experience of the item (Okamoto et al., 2004).

In contrast to fMRI, there are no prohibitions on metal for functional near infrared spectroscopy (fNIRS) and because the sensors are attached to the head and hence move with the head, it is relatively robust to movement (Piper et al., 2014). This is further evidenced by the proliferation of portable fNIRS devices (Pinti et al., 2018). This suggests that fNIRS may provide a complementary approach to fMRI by providing a means of consumer research involving actual use of a product. Because, the signal obtained *via* fNIRS is comparable to that of fMRI including for cognitive tasks activating frontal brain areas (Cui et al., 2011; Sato et al., 2013), fNIRS can in a sense serve as a real-world extension of previous fMRI studies.

Although relatively rare, there are published reports connecting brain activity recorded by fNIRS and commercially relevant outcomes. For example, frontal brain activity recorded by fNIRS during a task which involved thinking about the price of a product shown in a photograph along with simple product information, predicted the price later given by the participants (Misawa et al., 2014). In addition, fNIRS has also been used to assess brain activity during real use of a single skin care cream. The authors demonstrated that the fNIRS signal at various frontal channels correlated with the subjective rating of the cream (Nagai et al., 2012).

For fNIRS to be a viable method of assessing a product, it should differentiate and rank multiple products based on the recorded brain activation during real use of the products. Therefore, in the current study we investigated the possibility of using fNIRS to directly evaluate consumers’ purchase-related behavior, in this case WTP, during the real use of a product. Specifically, we focused on RH-DLPFC, more specifically the area previously reported as correlating with WTP (Plassmann et al., 2007), showing that brain activation in this region during real use of different cosmetic foundations correlates with their WTPs, within subjects.

## Materials and Methods

### Participants

After giving their informed consent, 30 female participants (average age: 21.2, SD: 0.86, range 20–22) were tested and remunerated for their time (JPY2000 book voucher). All participants were students in Chuo University.

As the participants were relatively young, their accumulated experience of foundation likely is highly dependent on the frequency with which they currently use foundation, in other words participants who use foundation almost every day would quickly have accumulated more experience and expertise than participants who do not use it every day. Therefore, based on their self-reported frequency of foundation usage, the participants were grouped into a higher frequency (HF) use group (*n* = 15), who used foundation 6 or more days a week and a lower frequency (LF) use group (*n* = 15), who used foundation 5 or less days a week. The experiment was conducted in the Applied Cognitive Neuroscience Laboratory in Chuo University, Bunkyo-ku, Tokyo during the first half of 2017 under the approval of the local ethics committees of both Chuo University and Shiseido Global Innovation Center. All subjects gave written informed consent in accordance with the Declaration of Helsinki.

### Foundations

Seven foundations were used in the experiment (see Table 1), and this included currently sold foundations and prototypes. We included a range of foundations and predicted that three would be judged to have a higher WTP, three would be judged to have a lower WTP, and the other foundation used for practice, which we predicted, should be of intermediate value. This prediction was based on the current market price for the foundation, product testing and characteristics of the foundation.

**Table 1:** Details of the seven foundations used including the ID by which there were referred to during the experiment and the predicted value.

ID	Predicted value
U	Lo
Q	Lo
R	Lo
P	Hi
S	Hi
T	Hi
W	Mid (practice)

*The value of prototype products was based on product testing and characteristics of the foundation. W was used only during practice*.

### Experimental Design

Before the experiment began, participants received a written and verbal explanation of the experiment and that the experiment was conditional on their informed consent, where there would be no disadvantage conferred upon them should they choose to not participate. During the experiment, participants sat upright in a chair in front of a three-paneled mirror, adjusted appropriately for height. The participants were unrestrained though they were encouraged to try to reduce rapid head movements, which may have introduced noise into the fNIRS.

**Figure 1:**
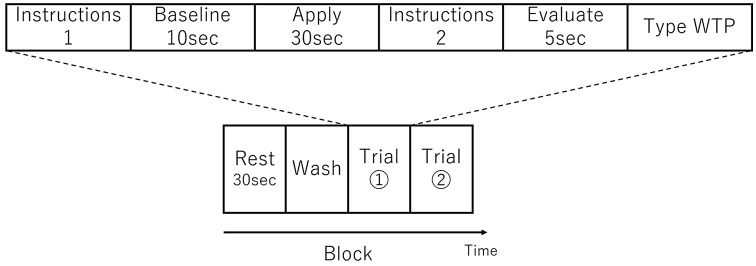
Schematic showing an experimental block, which consisted of two trials. An example trial is shown above the block schematic. The order of foundation and the side to which they were applied was randomized.

There were three sessions and each session comprised one or more blocks. A single block consisted of a *Rest* period, a *Wash* period, and two consecutive *Trials* (see Figure 1). In the *Rest* period, the participant was instructed to sit still for 30 s. In the *Wash* period, the participant wiped their face using a makeup-removing face wipe. After the participant had finished wiping their face, the first trial in the block started with the female experimenter handing the appropriate foundation to the participant on a cushion (typically used for foundation application) and then instructions were displayed on a computer monitor indicating which side of the face the foundation should be applied (Instructions 1 in Figure 1). When ready, the participant initiated a trial by pressing a key at which point the monitor displayed a blank white screen. Next, 10 s of baseline activity was recorded (*Baseline* in Figure 1). Following this an auditory cue indicated that the participant should begin applying the foundation (*Apply* in Figure 1). After 30 s, a second auditory cue indicated that the participant should stop the application. The monitor then displayed instructions (*Instructions 2* in Figure 1) for the participant to press the Enter key when ready, after which the monitor displayed a blank white screen and the participant should think about how much they would be willing to pay for the foundation (*Evaluate* in Figure 1). After 5 s, the monitor instructed the participant to enter their WTP value *via* a keyboard and press the Enter key (*Type WTP* in Figure 1). The participant was able to correct any typing mistakes they made using the Backspace key. The entry of the WTP was terminated by the participant pressing the Enter key. In other words, a single trial comprised the application to half face, evaluation and entering of WTP of a single foundation. The second trial in the block then started with the female experimenter handing the appropriate foundation to the participant on a cushion and the computer monitor indicating which side of the face the foundation should be applied (which in the second trial in a block was the opposite side to the first trial) and the trial proceeded in the same way as the first trial described above. The visual and auditory information were presented using E-Prime (E-Prime, Psychology Software Tools, Inc., Sharpsburg, PA, USA).

In the first session, in order to become familiar with the procedure, the participant had a “dry run” where they rehearsed a single block of the procedure without applying any foundation. In the second session, the participant completed a single block where they applied a typical mid-price range foundation (W in Table 1) to each side of their face. This functioned as a “wet run” practice. Next was the third, main session in which fNIRS brain imaging data was collected. In this session, the participant completed three blocks in which they applied three low price range and three high price range foundations (P-U in Table 1). Due to the number of conditions (six foundations, two face sides), it was not possible to counterbalance the order of foundation application. Instead, the order of foundation and the order of face side were randomized.

After the completion of the above three sessions, the participant completed a questionnaire about their foundation (see Supplementary Information) use as well as the Japanese version of the Edinburgh Handedness Inventory. Then finally, the position of the probe position was recorded using a 3D digitizer (POLHEMUS, Patriot).

### fNIRS Measurements

Brain hemodynamics were measured using the multichannel fNIRS system ETG-4000 (Hitachi Medical Corporation, Kashiwa, Japan), with two wavelengths of near-infrared light (695 and 830 nm). The modified Beer–Lambert Law (Cope et al., 1988) was used to analyze the optical data (Maki et al., 1995) and calculate signals reflecting the oxygenated hemoglobin (oxy-Hb), deoxygenated hemoglobin (deoxy-Hb), and total hemoglobin (total-Hb) signal changes, obtained in units of millimolar • millimeter (mM mm; Maki et al., 1995).

For statistical analyses, we focused on the oxy-Hb signal because of its higher sensitivity to changes in cerebral blood flow than that of deoxy-Hb and total-Hb signals (Hoshi et al., 2001; Strangman et al., 2002; Hoshi, 2003), its higher signal-to-noise ratio (Strangman et al., 2002), and its higher retest reliability (Plichta et al., 2006). However, we also include the analysis of the deoxy-Hb signal for completeness.

**Figure 2:**
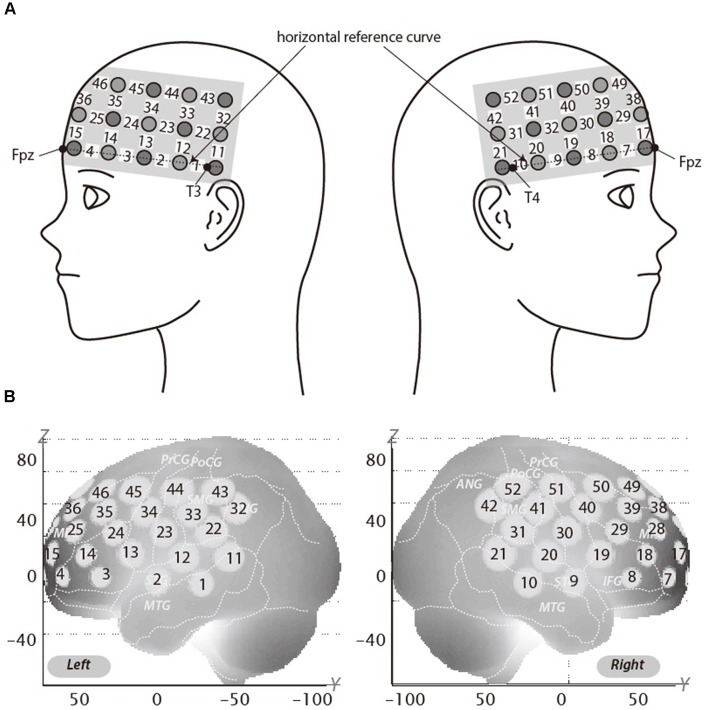
Spatial profiles of functional near infrared spectroscopy (fNIRS) channels. The upper panel **(A)** shows left and right side views of the probe arrangements are shown with fNIRS channel orientation. Detectors are indicated with dark circles, illuminators with light circles, and channels with white squares. Corresponding channel numbers are shown in black. The lower panel **(B)** shows the estimated channel locations on the brain for both left and right side views. The radii of the circles indicate the spatial variability associated with the estimation exhibited in the MNI space.

We used a 3 × 11 multichannel probe holders that consisted of 17 illuminating and 16 detecting probes arranged alternately at an inter-probe distance of 3 cm. This resulted in 52 channels (CH). We defined the midpoint of a pair of illuminating and detecting probes as a channel location. The fNIRS probes were placed such that Fpz coincided with the sixth probe in the middle column of holders in the 3 × 11 probe holder and the lower line substantially matched the horizontal reference curve, where the horizontal reference curve was determined by a straight line connecting Fpz–T3–T4. Thus, the probes covered our region of interest, dorsolateral PFC (DLPFC; Jurcak et al., 2007; Figure 2A).

### Spatial Profiling of fNIRS Data

For spatial profiling of fNIRS data (Figure 2B), we adopted virtual registration for registering fNIRS data to MNI standard brain space (Brett et al., 2002). Briefly, this method enables us to place a virtual probe holder on the scalp based on a simulation of the holder’s deformation and the registration of probes and channels onto reference brains in an MRI database (Okamoto and Dan, 2005; Okamoto et al., 2006). Accordingly, we estimated macroanatomical labels using a Talairach Daemon (Lancaster et al., 2000). DLPFC extends over a considerable area and it is likely that a number of channels recorded signal from this area. We therefore used the area of RH-DLPFC centered around the MNI coordinates which have been previously reported by Plassmann et al. (2007; *x* = 44, *y* = 44, *z* = 18) as correlating with WTP as our region of interest. According to the virtual registration, channel 39 was the closest channel to this location (Table 2). However, the results from other channels, including those which covered the greater DLPFC, are reported in Supplementary Materials.

**Table 2:** The MNI coordinates of channel 39 and the probability of the recorded data originating from the listed anatomical locations.

Channel	x	y	z	SD	Anatomy	Probability
39	47	37.33	36.33	9.647	9—RH Dorsolateral prefrontal cortex	0.624
					46—RH Dorsolateral prefrontal cortex	0.352
					8—RH Frontal eye fields	0.023

### Analysis of fNIRS Data

First, channels with signal variation of 10% or less were considered defective measurements and excluded from analysis. This corresponded to 2.23% of the data (no data from channel 39 was excluded). Then to remove the influence of measurement noise such as breathing, cardiac movement and so on from the remaining channels, wavelet minimum description length (Wavelet-MDL) was used (Jang et al., 2009).

Next the preprocessed oxy-Hb time-series data for each channel for each participant was analyzed in Matlab 2007b (The MathWorks, Inc., Natick, MA, USA) with the tools from Uga et al. (2014) using the adaptive GLM, by regressing the data with a linear combination of explanatory variables i.e., regressors and an error term. The regressors were created by convolving (Equation 2) the boxcar function N (τ_p,t_) with the hemodynamic response function (HRF) shown in Equation 1 (Friston et al., 1998). We set the first peak delay, τ_p_, 6 s as is commonly done, the second peak delay, τ_d_, was set to 16 s and A, the amplitude ratio between the first and second peak, was set to 6. The first and second derivatives were included in order to eliminate the influence of noise of individual data further. Equation 1: HRF.

(1) $$\text{h(}\tau_{\text{p}}\text{,t)=}\frac{{\text{t}}^{\tau_{\text{p}}}{\text{e}}^{-\text{t}}}{(\tau_{\text{p}})!}-\frac{{\text{t}}^{\tau_{\text{p}}+\tau_{\text{d}}}{\text{e}}^{-\text{t}}}{A(\tau_{\text{p}}+\tau_{d})!}$$

Equation 2: model waveform created by convolving the HRF and boxcar function.

(2) $$f(\tau_{\text{p}},\text{t})=h(\tau_{\text{p}},\text{t})*N(\tau_{\text{p}},\text{t})$$

**Figure 3:**
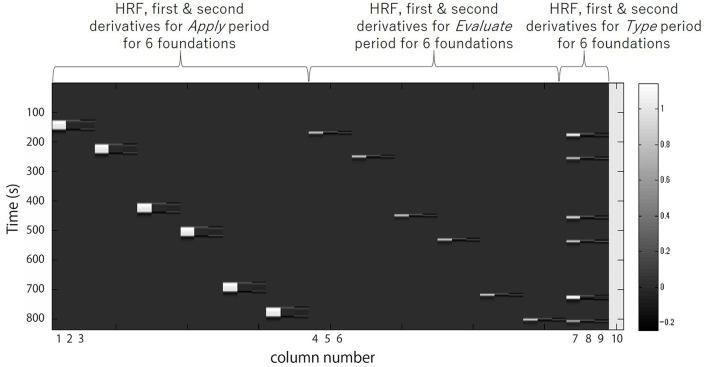
A example design matrix for the GLM model. The first peak delay was set as τ_p_ = 6 s and the row number represents the time sequence with time zero being at the top. The columns designated with 1, 2, and 3 indicate the canonical hemodynamic response function (HRF) f(τ_p,t_), the derivatives, and the second derivatives, respectively, for *Apply* period. There were six triplets of the regressors for application, representing six different samples. The columns designated with 4, 5, and 6 indicate those for *Evaluate* period. There were six triplets of the regressors for evaluation, representing six different samples. The column designated with 7, 8, and 9 indicate those for *Type* willingness-to-pay (WTP) period. The column indicated with 10 indicates the constant.

Regressors included were the 30 s *Apply*, the 5 s *Evaluate*, and the *Type WTP* for each trial. Columns 1, 2 and 3 in Figure 3 respectively represent the HRF of the *Apply* period and the first and second derivatives. Columns 4, 5 and 6 respectively represent the HRF of the *Evaluate* period and the first and second derivatives. Columns 7, 8 and 9 respectively represent the HRF of the *Type* *WTP* period and the first and second derivatives. Column 10 represents the constant. The β value is used as an estimate of the HRF prediction of the oxy-Hb signal. A total of six β values were calculated for both the *Apply* period (β_A1_, β_A2_, β_A3_, β_A4_, β_A5_, β_A6_) and the *Evaluate* period (β_E1_, β_E2_, β_E3_, β_E4_, β_E5_, β_E6_) and a single β value for the *Type WTP* period (β_type_), giving a total of 13 β values. β_A1_ is the β for the *Apply* period of the first foundation, β_E1_ the β for the *Evaluate* period of the first foundation and so on.

The procedure for the group level analysis is as follows. First, in order to test whether the different foundations caused different activation in RH-DLPFC and if this was affected by the frequency of use of foundation by the participant, the β value for each foundation was entered into a mixed analyses of variance (ANOVA) with foundation (U, Q, R, P, S, T) as the within subject factor and group (LF, HF) as the between subjects factor. Next, in order to investigate if the brain activation in RH-DLPFC could predict the WTP for the foundations a group averaged intrasubject correlation between WTP and RH-DLPFC activation was obtained as follows. The Spearman correlation coefficient between the *Apply* period beta values (βA1, βA2, βA3, βA4, βA5, βA6) obtained from channel 39 and the respective WTP values was calculated for each participant. The coefficient for each channel was then converted to a Z score using Fisher’s r-to-z transformation.

A one sample *t*-test was conducted to determine whether the average Z scores for each group (HF and LF) differed from 0 and then the two groups were compared *via* an independent *t*-test. Next, Fisher’s z-to-r transformation was used to convert the average Z for each group to a correlation coefficient.

The above process was repeated for the preprocessed deoxy-Hb time-series data.

## Results

### WTP Results

**Figure 4:**
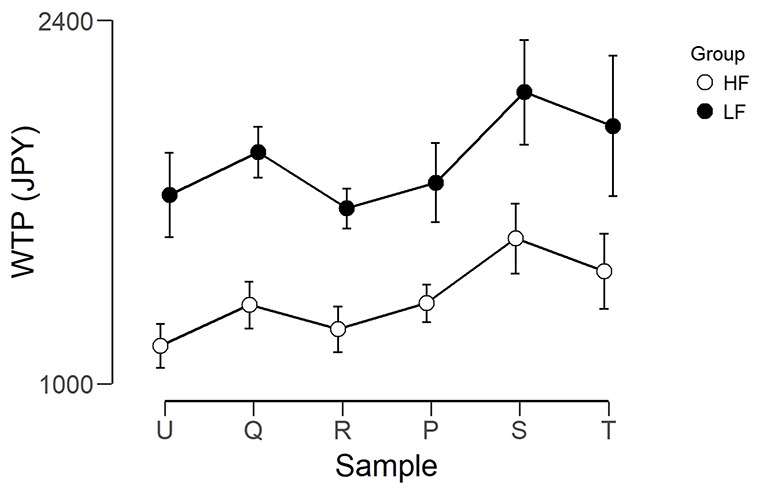
Graph showing the WTP in Japanese yen for each foundation for the two groups. Error bars represent the standard error of the mean. The left three foundations were predicted to be lower in value and the right three foundations were predicted to be higher in value.

Figure 4 shows the mean WTP for each foundation for the two groups. The WTP scores were analyzed using two mixed ANOVAs, using JASP Version 0.9 (JASP Team, 2018). The first had Group (LF, HF) as the between-subjects factor and Foundation (U, Q, R, P, S, T) as the within-subjects factor. The purpose of this ANOVA was to ensure there was meaningful variation between the different foundations, as without this, a correlation between WTP and brain activity would be impossible. There was a significant main effect of Foundation (*F*_(5,140)_ = 3.661, *p* = 0.004, η^2^ = 0.115). After correction for multiple comparisons, there was only a trend (*p* = 0.081) for a difference between R (mean = 1,443, SEM = 156) and S (mean = 1,842, SEM = 201). There was a trend for a main effect of Group (*F*_(1,28)_ = 3.345, *p* = 0.078, η^2^ = 0.107), where the WTP for the LF group were higher than those of the HF group. However, there was no interaction (*F*_(5,140)_ = 0.117, *p* = 0.988, η^2^ = 0.004). The second ANOVA had Group (LF, HF) as the between subjects factor and Predicted Price Range (Hi, Lo) as the within subjects factor (Hi was the average of the three foundations predicted to have a high price, and Lo was the average of the three foundations predicted to have a low price). There was a main effect of Predicted Price (*F*_(1,28)_ = 14.219, *p* < 0.001, η^2^ = 0.337) where Hi (mean = 1,699, SEM = 158.1) was greater than Lo (mean = 1,492, SEM = 152.0). The interaction between Predicted Price and Group was not significant.

### fNIRS Results: RH-DLPFC

**Figure 5:**
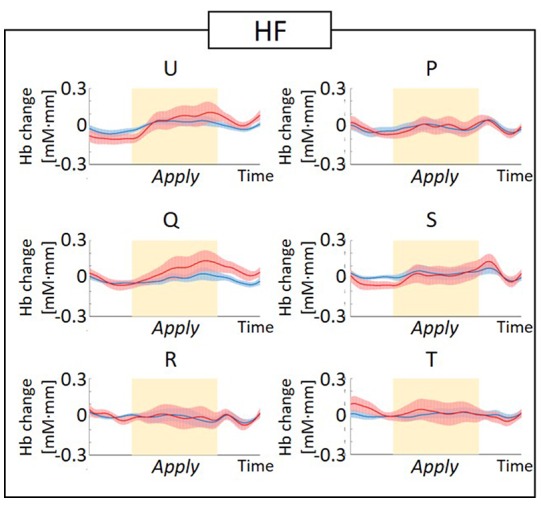
Graphs of the observed timeline data for fNIRS from right hemisphere dorsolateral prefrontal cortex (RH-DLPFC) for six foundations, averaged across the higher frequency (HF) group. The red lines indicate the observed timelines for oxygenated hemoglobin (oxy-Hb) signal and the blue lines indicate deoxygenated hemoglobin (deoxy-Hb) signal. Standard errors are shown as pale red (oxy-Hb) and blue (deoxy-Hb) areas. The yellow highlighted area is the *Apply* period.

**Figure 6:**
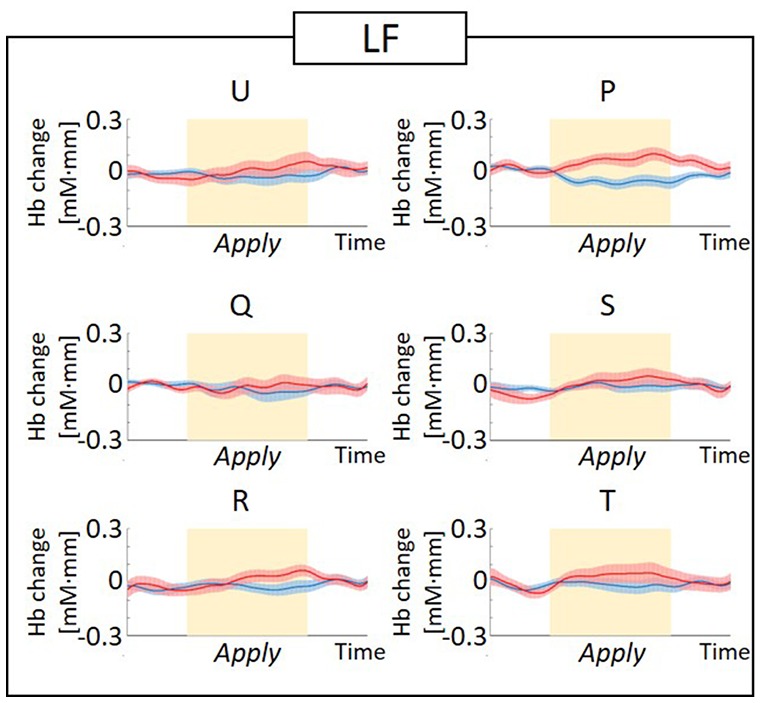
Graphs of the observed timeline data for fNIRS from RH-DLPFC for six foundations, averaged across the LF group. The red lines indicate the observed timelines for oxy-Hb signal and the blue lines indicate deoxy-Hb signal. Standard errors are shown as pale red (oxy-Hb) and blue (deoxy-Hb) areas. The yellow highlighted area is the *Apply* period.

**Figure 7:**
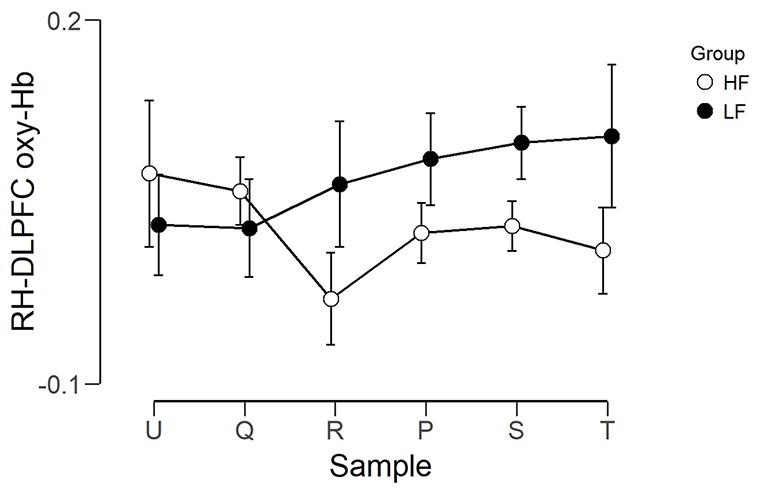
Graph showing the group average beta scores (oxy-Hb) for the six foundations for the two groups. Error bars represent the standard error of the mean.

**Figure 8:**
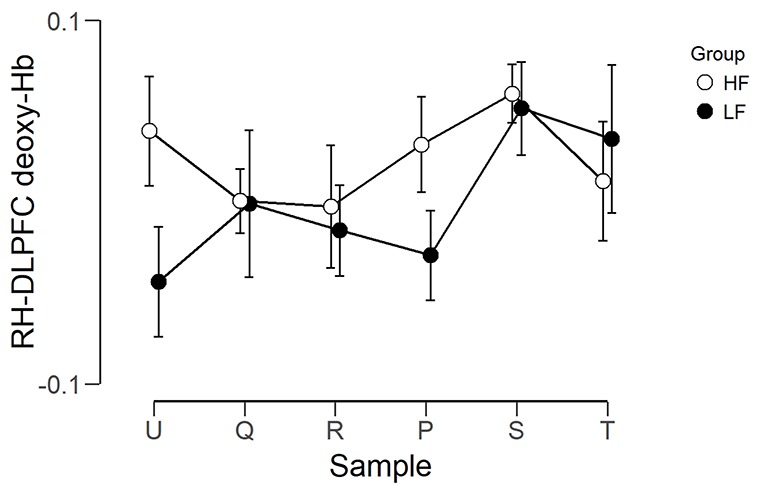
Graph showing the group average beta scores (deoxy-Hb) for the six foundations for the two groups. Error bars represent the standard error of the mean.

The observed timeline data for RH-DLPFC can be seen in Figures 5, 6. We investigated whether activation in the RH-DLPFC was related to either the foundation or the group in a mixed ANOVA with foundation as the within subjects factor (U, Q, R, P, S, T), and group (LF, HF) as the between subjects factor. For the oxy-Hb signal (see Figure 7), there was no main effect of sample (*F*_(3.635,101.767)_ = 0.331, *p* = 0.894, η^2^ = 0.011*), no main effect of group (*F*_(1,28)_ = 0.343, *p* = 0.563, η^2^ = 0.012) and no interaction (*F*_(3.635,101.767)_ = 1.135, *p* = 0.345, η^2^ = 0.039*). This was replicated in the deoxy-Hb signal (see Figure 8), where there was no main effect of sample (*F*_(3.489,97.689*)_ = 1.334, *p* = 0.262, η^2^ = 0.044), no main effect of group (*F*_(1,28*)_ = 0.208, *p* = 0.652, η^2^ = 0.007) and no interaction (*F*_(3.489,97.689)_ = 0.907, *p* = 0.453, η^2^ = 0.030; *As Mauchly’s test of sphericity indicated that the assumption of sphericity was violated, Greenhouse-Geisser’s correction was applied).

In other words, applying the different foundations did not result in significantly different activation in RH-DLPFC for all participants and this was unaffected by the frequency of foundation use.

**Figure 9:**
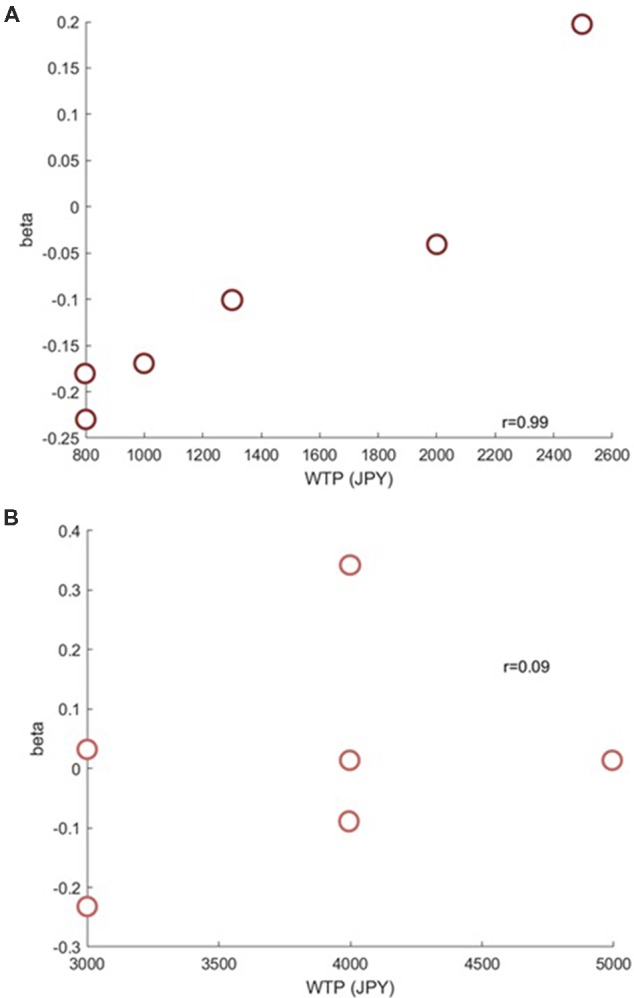
Example scatterplots from channel 39 (RH-DLPFC) from a participant in the HF group **(A)** and a participant in the LF group **(B)**. The positive correlation between RH-DLPFC activity and WTP in the HF participant is clear.

**Figure 10:**
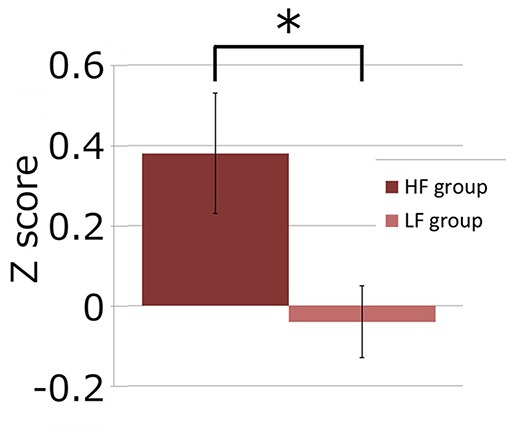
Graph showing channel 39 mean Z scores for the average correlation between brain activity and WTP for the two groups. The asterisk (*) indicates a significant difference (*p* < 0.05).

Next, we investigated if the brain activation in the RH-DLPFC could predict the WTP for the foundations at the individual level. Examples of the intra-subject correlations the for oxy-Hb signal are shown in Figure 9. As described in the methods, the correlation coefficient for each subject was transformed using Fisher’s z transformation into a Z score, to allow averaging across each group. After conversion to a Z score, a one sample *t*-test was conducted for each group to assess whether the group mean Z score was significantly different from 0. The HF group (mean = 0.384, SEM = 0.153) differed significantly from 0 (*t*_(14)_ = 2.504, *p* = 0.025, *d* = 0.646) whereas the LF group (mean = −0.037, SEM = 0.088) did not (*t*_(14)_ = 0.421, *p* = 0.680, *d* = 0.109). Furthermore, as can been seen in Figure 10, the HF group mean Z score was significantly higher than the LF group (*t*_(28)_ = 2.382, *p* = 0.024, *d* = 0.870). Finally, the mean Z scores for each group were converted to r scores using Fisher’s z transformation: the average correlation for HF was *r* = 0.366 and for LF was *r* = −0.037.

In contrast, for the deoxy-Hb signal, there was only a trend for the HF group mean Z score to differ from 0 (mean = 0.369, *t*_(14)_ = 2.002, *p* = 0.065, *d* = 0.517), while the LF group was not significantly different from 0 (mean = −0.033, *t*_(14)_ = 0.389, *p* = 0.702, *d* = 0.101). The two groups were not significantly different (*t*_(28)_ = 1.978, *p* = 0.058, *d* = 0.722).

## Discussion

In the current study, we showed that activation in the RH-DLPFC recorded with fNIRS during real use of variations of the same cosmetic product type correlates within a subject with the respective WTPs in consumers experienced in use of the cosmetic. To our knowledge, this is the first demonstration that fNIRS can detect neural correlates of WTP during a single use of a real product, not photograph, in a semi-naturalistic environment. In the current experiment, the participants sat comfortably in front of a mirror and could move freely to apply the product in a setting not dissimilar to their daily life. This further provides evidence that fNIRS can not only assist and enhance product development in a commercial setting but also be used to investigate brain function in naturalistic scenarios, complementing traditional lab-based research.

The evaluation of a cosmetic product by a consumer involves applying the product while observing its effect in a mirror and simultaneously monitoring the different properties of the product which the consumer considers determinants of value. For foundation, these may include visual properties such as tone, coverage, and cakeyness, and also somatosensory properties such as hydration, and texture. When calculating how much they are WTP for a particular foundation, the participant/consumer must first explore these physical properties of foundation, encoding them, and maintaining representations about the above properties in their working memory. Then, based on these properties and the interpretation thereof, they calculate a monetary value.

Previous neuroimaging and TMS studies have highlighted the role of RH-DLPFC in the encoding, retrieval and maintenance of nonverbal information (Kelley et al., 1998; Opitz et al., 2000; Okamoto et al., 2011; Rothmayr et al., 2007; Blanchet et al., 2010; Savini et al., 2012). For example, Kelley et al. (1998) reported intentional encoding of unfamiliar faces (that is, faces where the name is unknown) resulted in right lateralized DLPFC activity. Consistent with this, the correlation observed in the current study may reflect the engagement of RH-DLPFC by participants as they rely on nonverbal information to complete the evaluation.

The lack of correlation in the LF group may reflect the importance of learning and automation on RH-DLPFC activity (Jansma et al., 2001). It is likely that the experience that people who use foundation almost every day have accumulated allows them to expertly evaluate the product, including the texture, finish et cetera and match this experience more precisely to the price. This may have strengthened the relationship between the activation of RH-DLPFC in relation to the evaluation at the time of application and the WTP. In contrast, the relative inexperience of the LF group resulting in a “noisier” signal in RH-DLPFC rendering a correlation of a small number of data points difficult. This noise may reflect unspecific cognitive processing information not directly related to the task of deciding a WTP or simply neuronal noise which decreases *via* expertise (Gantz et al., 2007).

In contrast to the behavioral WTP, averaging the brain activity in RH-DLPFC for each product across participants did not reveal any differences between products. This may be because the brain signal recorded on a single trial lacks a sufficient signal-to-noise ratio. Rather the group average of an intra-subject correlation between WTP and brain activity revealed that the activation of RH-DLPFC of an individual participant reflected the value ascribed to a cosmetic product by that participant. This highlights the importance of considering the individual when investigating brain function. The intra-subject nature of the relationship between WTP and RH-DLPFC suggests the possibility that fNIRS may be used to provide an individual a personalized assessment of products. This combined with the ability of fNIRS to investigate brain responses in naturalistic settings, suggests that fNIRS may have a role to play in the spread of mass-personalization of products and services^1^.

## Author Contributions

KKD, CS, KT and ID designed the research. KKD, TT, CS performed the experiment. KKD, TT, CS and ID analyzed the data. KKD drafted the work. KT and ID revised the manuscript.

## Conflict of Interest Statement

KKD, CS and KT are employed by Shiseido Global Innovation Center, Shiseido Co., Ltd., a company which manufactures cosmetics. KKD, CS, KT, TT and ID are named inventors on a patent application covering the method disclosed in this research.

## Footnotes

^1^Deloitte Global. (n.d.). *Made-to-order: The rise of mass personalization*. Retrieved September 12, 2018, from: https://www2.deloitte.com/content/dam/Deloitte/tr/Documents/consumer-business/consumer-business-mass-personalisation.pdf

## Supplementary Material

The Supplementary Data are shared on FigShare here: https://doi.org/10.6084/m9.figshare.7409834.v4
