# Supplementary material for: Willingness-to-Pay-Associated Right Prefrontal Activation During a Single, Real Use of Cosmetics as Revealed by Functional Near-Infrared Spectroscopy
Source: Front Hum Neurosci. 2019 Feb 4;13:16. doi: 10.3389/fnhum.2019.00016 (PMC6369365; doi:10.3389/fnhum.2019.00016)
Supplement: Supplementary file 1 [file Data_Sheet_1.docx]

Supplementary Material

Willingness-to-pay-associated right prefrontal activation during a single, real use of cosmetics as revealed by functional near-infrared spectroscopy

**Keith Kawabata Duncan^1*^, Tatsuya Tokuda^2^, Chiho Sato^1^, Keiko Tagai^1^, Ippeita Dan^2^**

^1^Shiseido Global Innovation Center, 2-1-1, Hayabuchi, Tsuzuki-ku, Yokohama, Kanagawa, 224-8558, Japan

^2^Chuo University, Applied Cognitive Neuroscience Laboratory, 1-13-27 Kasuga, Bunkyo, Tokyo, 112-8551, Japan

***Correspondence:**Dr. Keith Kawabata Duncan

[keith.kawabata.duncan@to.shiseido.co.jp](mailto:keith.kawabata.duncan@to.shiseido.co.jp)

# Supplementary Information

## Final questionnaire

**Q1. Please enter how you made the decision regarding about how much you were willing to pay for the foundations.**

Please complete the following.

**Q2．What foundation do you typically use?**

**Brand:**

**Product:**

**Color code:**

**Type:**

- Powdery
- Liquid
- Cream
- Cushion
- BB cream

< If you responded “Powdery” in Q2, please go to Q4. >

**Q3. Have you ever used powdery foundation?**

- I have used it on a daily basis.
- I have tried it
- I have never used it

**Q4. What is the price of the foundation entered in Q2?**

- JPY1000 or less
- JPY1001 to JPY2000
- JPY2001 to JPY3000
- JPY3001 to JPY4000
- JPY4001 to JPY5000
- JPY5001 or more

**Q5. How frequently do you use foundation?**

- 1 day or less per week
- 2 to 3 days per week
- 4 to 5 days per week
- 6 to 7 days per week

**Q6. Please enter your age**

## Final questionnaire results

**Q1.** Answers to this question can be grouped into the following five reasons.

- Covering redness, blemishes, and pores or not
- Sheer coverage or full coverage
- Blending into skin or not
- Smoothness of spreading
- Color, light or dark shade

**Q2.** A variety of domestic and international brands were listed

**Q3.** Three participants reported they had never tried powdery foundation (1 in the LF group and 2 in the HF group). However, for an unknown reason, 15 participants did not answer this question.

**Q4.** The results of Q4 can be seen in the following pie chart.


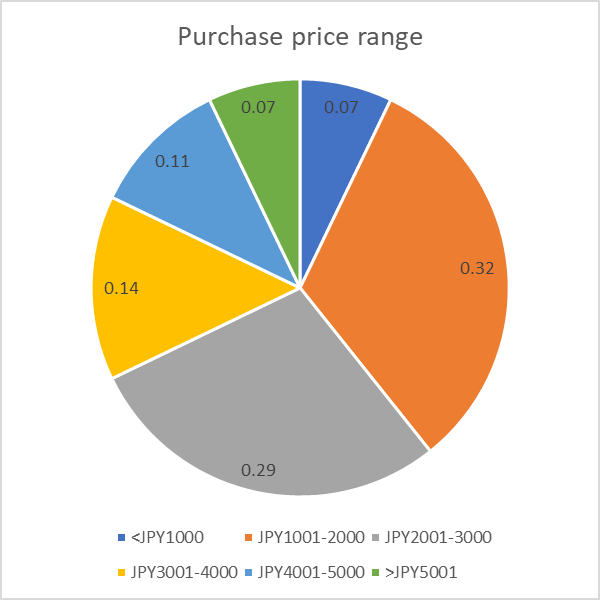


Two participants failed to give an answer to this question.

**Q5.** This question was used to group the participants in to HF and LF groups.


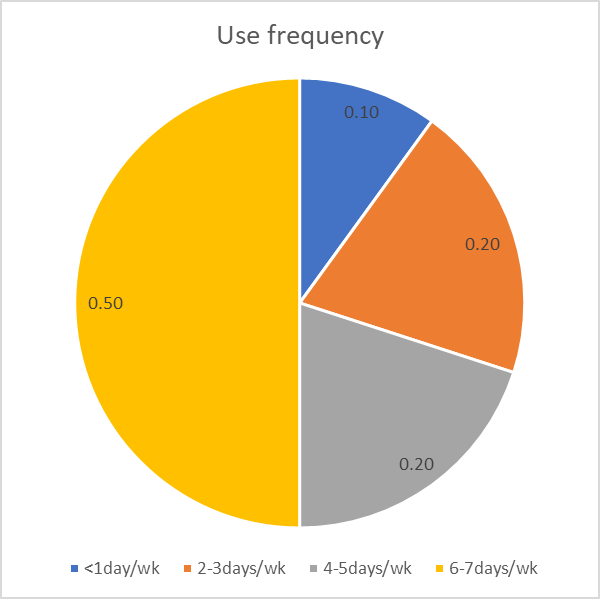


**Q6.** As reported in the methods, the average age was 21.2, *SD*: 0.86, range 20-22. There was no difference between the ages of the HF group (mean = 21.33, SD = 0.900) and the LF group (mean = 21.133, SD = 0.834; *t*(28) = 0.631, *p* = 0.533, d = 0.231).

Table S1: Results of one sample t tests for HF and LF groups, and the results of the two samples t test of the comparison of the correlations of HF and LF groups.

|  | HF group | | | | | LF group | | | | | HF vs LF | | |
| --- | --- | --- | --- | --- | --- | --- | --- | --- | --- | --- | --- | --- | --- |
| channel | Mean z | t | df | p | Mean r | Mean z | t | df | p | Mean r | t | df | p |
| 1 | -0.04 | -0.28 | 14 | 0.79 | -0.04 | -0.11 | -0.82 | 14 | 0.43 | -0.11 | 0.34 | 28 | 0.74 |
| 2 | -0.13 | -1.21 | 14 | 0.25 | -0.13 | -0.01 | -0.04 | 13 | 0.97 | -0.01 | -0.62 | 27 | 0.54 |
| 3 | 0.16 | 0.88 | 13 | 0.39 | 0.16 | -0.18 | -1.31 | 13 | 0.21 | -0.17 | 1.49 | 26 | 0.15 |
| 4 | -0.05 | -0.50 | 14 | 0.63 | -0.05 | -0.19 | -2.13 | 14 | 0.05 | -0.19 | 1.04 | 28 | 0.31 |
| 5 | -0.18 | -1.11 | 14 | 0.29 | -0.17 | -0.09 | -0.44 | 14 | 0.67 | -0.09 | -0.34 | 28 | 0.74 |
| 6 | -0.07 | -0.35 | 13 | 0.73 | -0.07 | -0.09 | -0.85 | 14 | 0.41 | -0.09 | 0.13 | 27 | 0.90 |
| 7 | 0.30 | 2.26 | 14 | 0.04 | 0.29 | -0.18 | -2.18 | 14 | 0.05 | -0.17 | 3.06 | 28 | 0.01 |
| 8 | 0.10 | 0.56 | 13 | 0.59 | 0.10 | -0.01 | -0.07 | 14 | 0.94 | -0.01 | 0.44 | 27 | 0.67 |
| 9 | 0.04 | 0.32 | 13 | 0.75 | 0.04 | -0.03 | -0.21 | 12 | 0.84 | -0.03 | 0.37 | 25 | 0.71 |
| 10 | 0.02 | 0.18 | 13 | 0.86 | 0.02 | -0.04 | -0.24 | 13 | 0.82 | -0.04 | 0.29 | 26 | 0.77 |
| 11 | -0.09 | -0.64 | 13 | 0.53 | -0.09 | -0.18 | -1.37 | 14 | 0.19 | -0.18 | 0.47 | 27 | 0.65 |
| 12 | -0.12 | -0.66 | 14 | 0.52 | -0.12 | -0.04 | -0.25 | 13 | 0.81 | -0.04 | -0.33 | 27 | 0.74 |
| 13 | -0.05 | -0.38 | 14 | 0.71 | -0.05 | -0.07 | -0.53 | 13 | 0.61 | -0.07 | 0.10 | 27 | 0.92 |
| 14 | -0.07 | -0.33 | 13 | 0.74 | -0.07 | -0.05 | -0.41 | 13 | 0.69 | -0.05 | -0.11 | 26 | 0.92 |
| 15 | -0.30 | -1.55 | 14 | 0.14 | -0.29 | -0.22 | -2.29 | 14 | 0.04 | -0.22 | -0.39 | 20.271 | 0.70 |
| 16 | -0.12 | -0.67 | 14 | 0.51 | -0.12 | 0.02 | 0.22 | 14 | 0.83 | 0.02 | -0.70 | 28 | 0.49 |
| 17 | -0.11 | -0.78 | 14 | 0.45 | -0.11 | -0.01 | -0.10 | 14 | 0.92 | -0.01 | -0.67 | 19.28 | 0.51 |
| 18 | 0.10 | 0.51 | 14 | 0.62 | 0.10 | -0.18 | -1.80 | 14 | 0.09 | -0.18 | 1.27 | 28 | 0.21 |
| 19 | 0.02 | 0.12 | 13 | 0.91 | 0.02 | -0.02 | -0.15 | 14 | 0.88 | -0.02 | 0.19 | 27 | 0.85 |
| 20 | 0.23 | 1.44 | 14 | 0.17 | 0.23 | 0.08 | 0.66 | 13 | 0.52 | 0.08 | 0.74 | 27 | 0.47 |
| 21 | 0.38 | 2.02 | 14 | 0.06 | 0.36 | -0.07 | -0.52 | 13 | 0.61 | -0.07 | 1.93 | 27 | 0.06 |
| 22 | 0.05 | 0.30 | 13 | 0.77 | 0.05 | 0.00 | 0.01 | 14 | 0.99 | 0.00 | 0.19 | 27 | 0.85 |
| 23 | -0.04 | -0.22 | 14 | 0.83 | -0.04 | 0.08 | 0.36 | 14 | 0.73 | 0.08 | -0.41 | 28 | 0.68 |
| 24 | 0.14 | 0.63 | 13 | 0.54 | 0.14 | -0.04 | -0.26 | 13 | 0.80 | -0.04 | 0.67 | 26 | 0.51 |
| 25 | -0.08 | -0.39 | 14 | 0.70 | -0.08 | -0.09 | -0.75 | 13 | 0.47 | -0.09 | 0.07 | 27 | 0.95 |
| 26 | -0.13 | -0.81 | 14 | 0.43 | -0.12 | -0.04 | -0.33 | 14 | 0.74 | -0.04 | -0.49 | 28 | 0.63 |
| 27 | 0.17 | 0.97 | 14 | 0.35 | 0.17 | -0.06 | -0.65 | 14 | 0.52 | -0.06 | 1.16 | 21.565 | 0.26 |
| 28 | 0.03 | 0.18 | 14 | 0.86 | 0.03 | -0.12 | -1.01 | 14 | 0.33 | -0.12 | 0.74 | 28 | 0.47 |
| 29 | 0.07 | 0.61 | 14 | 0.55 | 0.07 | 0.09 | 0.98 | 14 | 0.34 | 0.09 | -0.15 | 28 | 0.88 |
| 30 | 0.26 | 1.89 | 14 | 0.08 | 0.25 | -0.10 | -0.71 | 14 | 0.49 | -0.10 | 1.83 | 28 | 0.08 |
| 31 | 0.09 | 0.87 | 14 | 0.40 | 0.09 | -0.18 | -1.28 | 14 | 0.22 | -0.17 | 1.55 | 28 | 0.13 |
| 32 | -0.03 | -0.17 | 13 | 0.87 | -0.03 | 0.07 | 0.56 | 13 | 0.59 | 0.07 | -0.45 | 26 | 0.66 |
| 33 | -0.28 | -1.80 | 14 | 0.09 | -0.27 | 0.21 | 1.11 | 14 | 0.29 | 0.21 | -1.99 | 28 | 0.06 |
| 34 | 0.40 | 1.88 | 14 | 0.08 | 0.38 | -0.01 | -0.10 | 13 | 0.92 | -0.01 | 1.62 | 27 | 0.12 |
| 35 | 0.03 | 0.16 | 13 | 0.87 | 0.03 | 0.07 | 0.42 | 13 | 0.68 | 0.07 | -0.16 | 26 | 0.88 |
| 36 | 0.12 | 1.01 | 14 | 0.33 | 0.12 | -0.08 | -0.69 | 14 | 0.50 | -0.08 | 1.20 | 28 | 0.24 |
| 37 | 0.31 | 1.33 | 14 | 0.20 | 0.30 | -0.09 | -0.84 | 14 | 0.42 | -0.09 | 1.56 | 19.483 | 0.14 |
| 38 | 0.29 | 1.60 | 14 | 0.13 | 0.28 | -0.25 | -2.23 | 14 | 0.04 | -0.24 | 2.53 | 23.37 | 0.02 |
| 39 | 0.38 | 2.50 | 14 | 0.03 | 0.37 | 0.03 | 0.22 | 14 | 0.83 | 0.03 | 1.78 | 28 | 0.09 |
| 40 | 0.08 | 0.52 | 13 | 0.62 | 0.08 | -0.13 | -1.53 | 14 | 0.15 | -0.13 | 1.24 | 27 | 0.23 |
| 41 | 0.06 | 0.54 | 14 | 0.60 | 0.06 | -0.13 | -1.10 | 14 | 0.29 | -0.13 | 1.17 | 28 | 0.25 |
| 42 | 0.09 | 0.54 | 14 | 0.60 | 0.09 | 0.01 | 0.08 | 14 | 0.94 | 0.01 | 0.39 | 28 | 0.70 |
| 43 | -0.34 | -2.63 | 14 | 0.02 | -0.33 | -0.04 | -0.60 | 13 | 0.56 | -0.04 | -2.03 | 21.451 | 0.06 |
| 44 | -0.06 | -0.50 | 14 | 0.62 | -0.06 | -0.19 | -1.90 | 14 | 0.08 | -0.19 | 0.86 | 28 | 0.40 |
| 45 | 0.13 | 0.72 | 14 | 0.48 | 0.13 | -0.13 | -0.79 | 13 | 0.44 | -0.13 | 1.06 | 27 | 0.30 |
| 46 | 0.00 | 0.02 | 14 | 0.98 | 0.00 | -0.28 | -2.24 | 14 | 0.04 | -0.27 | 1.15 | 28 | 0.26 |
| 47 | 0.06 | 0.28 | 13 | 0.79 | 0.06 | -0.03 | -0.25 | 14 | 0.81 | -0.03 | 0.37 | 27 | 0.71 |
| 48 | 0.30 | 2.27 | 14 | 0.04 | 0.29 | -0.14 | -0.61 | 14 | 0.55 | -0.14 | 1.68 | 28 | 0.10 |
| 49 | -0.07 | -0.41 | 14 | 0.69 | -0.07 | -0.15 | -1.20 | 14 | 0.25 | -0.15 | 0.38 | 28 | 0.71 |
| 50 | 0.10 | 0.59 | 14 | 0.56 | 0.10 | -0.31 | -2.87 | 13 | 0.01 | -0.30 | 1.99 | 27 | 0.06 |
| 51 | -0.05 | -0.38 | 14 | 0.71 | -0.05 | -0.41 | -3.01 | 12 | 0.01 | -0.39 | 1.81 | 26 | 0.08 |
| 52 | -0.18 | -1.67 | 14 | 0.12 | -0.18 | -0.06 | -0.69 | 14 | 0.50 | -0.06 | -0.85 | 28 | 0.40 |

## fNIRS results: Other brain areas

We had no a priori hypotheses about the relationship between WTP and other channels. However, for completeness the mean z values and results of one- and two-sample t tests (uncorrected) are shown in Supplementary Information Table 1 and the channels with a mean z value significantly greater than 0 are detailed below. All significant positive correlations were for the HF group whereas all significant correlations for the LF were negative.

#### Channel 7 (right fronto-polar area)

For channel 7, the mean z value for the HF group was positive (mean = 0.301, sem = 0.133) and differed significantly from 0 (t(14) = 2.26, p = 0.004, d = 0.0.584) whereas the mean z value for the LF group was negative (mean = -0.175, sem = 0.08) and differed significantly from 0 (t(14) = 2.184, p = 0.046, d = 0.546), and the two groups differed significantly from each other (t(22.954) = 3.064, p = 0.005, d = 1.119). In other words, as WTP increases, the activity in channel 7 increases for the HF group but decreases for the LF group. According to the result of the registration, channel 7 corresponds to the frontopolar area and orbitofrontal area (Table 3).

#### Channel 43 (left supramarginal gyrus)

For channel 43, the mean z value for the HF group was negative (mean = -3.444, sem = 0.131) and differed significantly from 0 (t(14) = 2.633, p = 0.020, d = 0.680). In contrast, the LF group mean z value (mean = -0.396, sem = 0.066) did not differ from 0 (t(14) = 0.599, p = 0.559, d = 0.160). The two groups differed significantly from each other (t(20.715) = 2.080, p = 0.050, d = 0.739, corrected for non-homogeneity). According to the result of the registration, channel 43 corresponds to the LH supramarginal gyrus area and primary somatosensory cortex (Table 3).

#### Channel 48 (right superior frontal gyrus)

For channel 48, the mean z value for the HF group was positive (mean = 0.302, sem = 0.133) and differed significantly from 0 (t(14) = 2.270, p = 0.040, d = 0.586). In contrast, the LF group mean z value (mean = -0.136, sem = 0.224) did not differ from 0 (t(14) = 0.607, p = 0.553, d = 0.157). However, the two groups did not differ significantly from each other (t(28) = 1.682, p = 0.104, d = 0.614). According to the result of the registration, channel 48 corresponds to the RH frontal eye fields (superior frontal gyrus) and RH-DLPFC (Table 3).

Table S2: The MNI coordinates of other channels with a significant correlation for the HF group, and the probability of the recorded data originating from the listed anatomical locations.

| Channel | x | y | z | SD | Anatomy | Probability |
| --- | --- | --- | --- | --- | --- | --- |
| 7 | 41.66 | 62.33 | -6.33 | 8.253 | 10 – RH Frontopolar area | 0.756 |
|  |  |  |  |  | 11 – RH Orbitofrontal area | 0.244 |
| 43 | -64.33 | -40.33 | 43.66 | 11.861 | 40 – LH Supramarginal gyrus | 0.958 |
|  |  |  |  |  | 2 – LH Primary somatosensory cortex | 0.042 |
| 48 | 9.33 | 46.66 | 53.33 | 12.331 | 8 – RH frontal eye fields | 0.944 |
|  |  |  |  |  | 9 – RH Dorsolateral prefrontal cortex | 0.056 |
